# Effects of Different Microbial Fertilizers on Growth and Rhizosphere Soil Properties of Corn in Newly Reclaimed Land

**DOI:** 10.3390/plants11151978

**Published:** 2022-07-29

**Authors:** Xuqing Li, Qiujun Lu, Dingyi Li, Daoze Wang, Xiaoxu Ren, Jianli Yan, Temoor Ahmed, Bin Li

**Affiliations:** 1 Institute of Vegetable, Hangzhou Academy of Agricultural Sciences, Hangzhou 310024, China; lixqing@hz.cn (X.L.); renxxhz@gmail.com (X.R.); 2 Hangzhou Agricultural and Rural Affairs Guarantee Center, Hangzhou 310020, China; 3 Department of Biological Environment, Material and Environmental College, Shanxi Jinzhong Institute of Technology, Jinzhong 030600, China; wangdc@hz.cn; 4 Rural Vitalization Service Center of Hangzhou, Hangzhou 310020, China; wdz2005@hz.cn; 5 Institute of Biotechnology, Zhejiang University, Hangzhou 310058, China; temoorahmed@zju.edu.cn (T.A.); libin0571@zju.edu.cn (B.L.)

**Keywords:** corn, land, microbial fertilizer, soil, microbiome

## Abstract

Land reclamation may expand the supply of usable land for food security. Soil microorganisms have been considered as an amendment in immature soil to improve its quality. However, different microbial fertilizers’ effects on plant growth in immature soil have largely been unexplored. In order to evaluate the effects of different microbial fertilizers on immature soil, the soil quality and microbial community structure of corn rhizosphere soil samples under different microbial fertilizers were investigated. The results revealed a significant difference between microbial fertilizers (especially seaweed microbial fertilizer, SMF) and commercial chemical compound fertilizers in the soil properties and microbial community structure. Indeed, SMF caused a 486.21%, 23.17%, 21.08%, 38.33%, and 482.39% increase in *Flavobacteriaceae*, *Planctomycetaceae*, *Chitinophagaceae*, *Acidobacteria_Gp3*, and *Mortierellaceae* but a 23.82%, 18.66%, 42.36%, 29.12%, 81.97%, 42.19%, and 99.33% reduction in *Cytophagales*, *Comamonadaceae*, *Rhodospirillaceae*, *Sinobacteaceae*, *Aspergillaceae*, *Myrmecridiaceae*, and *Typhulaceae*, respectively; while CCF caused an 85.68% and 183.22% increase in *Xanthomonadaceae* and *Mortierellaceae* but a 31.29%, 36.02%, and 65.74% reduction in *Cytophagales*, *Spartobacteria*, and *Cyphellophoraceae* compared with the control based on 16S and ITS amplicon sequencing of soil microflora. Furthermore, redundancy discriminant analysis of the microbial communities and soil properties indicated that the main variables of the bacterial and fungal communities included exchangeable Ca, organic matter content, total N, and available P. Overall, the results of this study revealed significant changes under different fertilizer conditions in the microbiota and chemical properties of corn soil. Microbial fertilizers, particularly SMF and SM, can be used as a good amendment for newly reclaimed land.

## 1. Introduction

China is one of the largest developing countries in the world. The very large population and little arable land are China’s national conditions, and food security is a huge problem currently facing the country [1]. In the past several years, with the development of urbanization and industrialization, the arable area and available land resources have decreased. In order to meet the demand for land, the barren mountain land and forest land in the mountainous areas of Zhejiang Province, China, were reclaimed for agricultural use [2]. However, in most situations, the quality of newly reclaimed land is not consistent with the quality of the occupied cultivated land and is not suitable for plant growth because it is acidic and poor in nutrients with a high gravel content [3]. For example, Hu et al. [1] reported that the production capacity of newly reclaimed land is only 10–30% of the occupied cultivated land in Hangzhou of Zhejiang Province, China [1]. So, a balance of occupation and compensation has been proposed in China, including both quantity and quality.

It is well known that all the factors affecting soil properties, including physical, chemical, and biological properties, could impact on soil quality [4,5,6]. Previous research has shown that the quality of newly reclaimed land can be effectively improved by increasing the use of organic fertilizer, such as biosolids, crop residues, livestock manure, and so on, which can not only modify soil physical and chemical properties but also exert a great influence on microbial communities [7]. Moreover, soil microorganisms have been reported to play an important role in the evolution of soil ecosystems and nutrient transformation, such as the production of siderophores, phosphate solubilization, and nitrogen fixation [8,9,10,11], and the application of plant growth-promoting microorganism-based biofertilizers to soil is considered as an eco-friendly, inexpensive, and sustainable alternative to hazardous chemical fertilizers [12]. However, although Li et al. (2021) found that four fungi isolates and five bacteria strains have great potential in promoting eggplant growth in immature soil obtained from newly reclaimed land [13,14], there is very little information available on the effect of microbial fertilizers on plant growth in the immature field.

The objective of this study was to evaluate the effect of different microbial fertilizers on corn growth in newly reclaimed land and the rhizosphere soil properties and microbial community structure. In addition, we examined the correlation between soil properties and the microbial community structure. This study provides a scientific basis to develop an effective measure to improve the soil quality of newly reclaimed land in the future.

## 2. Materials and Methods

### 2.1. Experimental Design

This experiment was carried out on newly reclaimed land in Baijiang Village (29°50′7″ N; 119°20′56″ E, 85 m) of Tonglu City, Zhejiang Province, China. The soil is silty clay loam, based on the soil classification system of the FAO-UNESCO, while its fertility grade (1–10, with 1 being the best) is grade 3 and class 6 based on the nutrient classification standard of the second soil survey in China. The top 20-cm soils have a pH of 6.45, with 0.072 g/kg of total N, 12.56 g/kg of organic matter contents, 13.80 mg/kg of available P, and 358 mg/kg of available K.

The experiment consisted of six different treatments through the application of various microbial/organic/chemical fertilizers to newly reclaimed land. Three kinds of microbial organic fertilizer, including Tuzangjin microbial fertilizer (TMF, provided by Beijing Yufengliduojin fertilizer Co., Ltd., Beijing, China), carbonergic microbial agent (CMA, provided by Shike Biotechnology (Shanghai) Co., Ltd., Shanghai, China), and seaweed microbial fertilizer (SMF, Qingdao Lvbaozhu biological Group Co., Ltd., Qingdao, China), were selected and used in this study. In detail, 0.825 kg of TMF, 1.50 kg of CMA, and 1.35 kg of SMF were applied to each m^2^ of the experimental field, respectively. Meanwhile, 1.50 kg of sheep manure (SM, Hangzhou Nanwuzhuang Soil Fertilizer Co., Ltd., Hangzhou, China) and 0.075 kg of commercial chemical compound fertilizer (CCF, Shenzhen Batian Ecological Engineering Co., Ltd., Shenzhen, China) (N-P-K, 16-16-16) were applied per m^2^ of experimental field, respectively. The treatment without any fertilizer was used as the control. The composition of each soil amendment used in this study is shown in Table 1.

**Figure 1:**
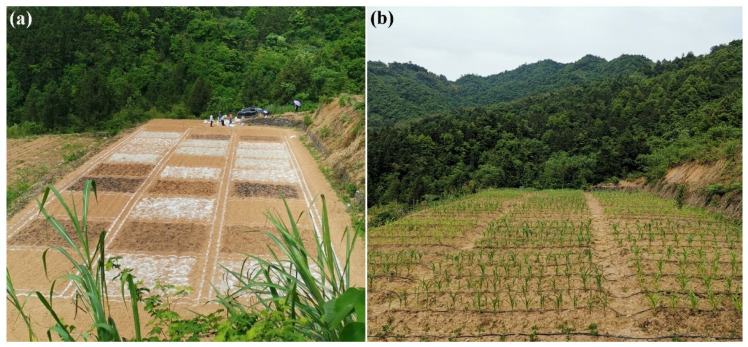
Experimental site for the evaluation of different fertilizers on the growth of corn in newly reclaimed land. (**a**) Soil with different fertilizers; (**b**) plantation of corn.

The field plot experiment was completely randomly designed in this study and was conducted for 3 years. The area of each plot was 20 m^2^ and the width and depth of the plot separated by the ditch were 30 and 20 cm, respectively (Figure 1). Each year, the top 0–20-cm soil of the experimental field was mixed with different fertilizers before sowing. The seeds of corn (cultivar ‘Zhongjiangyu 99’, Jiangsu Zhongjiang Seed Industry Co., Ltd., Suqian, China) were sown in the above-mentioned newly reclaimed land treated by different fertilizers. The treatment without any fertilizers was applied as the control. The experiment was carried out from April to August each year while the ripe ears of corn were harvested about 110 days after planting. Each treatment had three replicates.

### 2.2. Measure of Soil and Corn Production

In order to evaluate the effects of different microbial fertilizers on the plant growth and rhizosphere soil properties of corn in the newly reclaimed land, the ripe ears of corn and soil samples were collected about 110 days after planting and the weight of air-dried corn ears was measured by a digital scale (TCS-50, Shanghai hento Industrial Co., Ltd., Shanghai, China). The soil properties, including the pH, organic matter content, total N, available P, available K, exchangeable Ca, and exchangeable Mg, were detected as described in [15]. In detail, when corn was collected each year, about 1.0 kg of fresh soil samples from the rhizosphere soil (5–20 cm) of each plot were collected using the quartering method and a shovel with a scale and then passed through a 0.45-mm sieve to remove fine roots and debris. The soil properties were measured after the sample was dried at room temperature. Briefly, the pH of the soil was measured at a soil/distilled water suspension ratio of 1:5 (g/mL) with a pH meter (FE28, MettlerToledo, Zurich, Switzerland); the content of organic matter was determined by the K_2_Cr_2_O_7_ oxidation external heating method; the content of total N was determined using an automatic Kjeldahl distillation-titration unit; the content of available P was determined by hydrochloric acid–ammonium fluoride extraction molybdenum–antimony anti–colorimetry; the available K was extracted by ammonium acetate, and the contents were determined by a flame photometer; and exchangeable Ca and exchangeable Mg were extracted by ammonium acetate, and the contents were determined by an iCE 3500 atomic absorption spectrophotometer. All the treatments had three replicates.

### 2.3. Soil Genome Sequencing and Analysis

#### 2.3.1. Collection of Rhizosphere Samples and DNA Extraction

In total, 20 g rhizosphere soil of corn was sampled in the third year (in 2021). The DNA extraction of the soil samples was carried out according to the manufacturer’s instructions using the E.Z.N.A^TM^ Mag–Bind Soil DNA Kit (OMEGA, Norcross, GA, USA). The quality of extracted DNA was determined using a NanoDrop (ND-1000) spectrophotometer (ThermoFisher Scientific, Wilmington, DE, USA).

#### 2.3.2. Soil Genome Sequencing

The bacterial diversity was evaluated by the 16S rRNA V3–V4 region, amplified with the primers 341F (5′–CCTACGGGNGGCWGCAG–3′) and 805R (5′–GACTACHVGGGTATCTAATCC–3′) [16], whereas, for fungal diversity, the ITS1 and ITS2 region was amplified using the primers ITS1F (5′–CTTGGTCATTTAGGAAG TAA–3′) and ITS2 (5′–GCTGCGTTCTTCATTCGATGC–3′) [17], respectively. The components of the PCR included 12 μL ddH_2_O, 15 μL 2 × Hieff^®^ Robust PCR Master Mix, 1 μL DNA template, and 1 μL (10 μM) each forward and reverse primer. The PCR amplicons were purified with V azyme V AHTSTM DNA clean beads (Vazyme, Nanjing, China). Afterward, amplicons with equal amounts were pooled and 2 × 250-bp pair-end sequencing was accomplished through the Illumina MiSeq system (Sangon Biotech (Shanghai) Co., Ltd., Shanghai, China).

### 2.4. Statistical Analysis

The bioinformatics analysis of the microbiome was accomplished according to our previous study [18]. In brief, sequence raw data were demultiplexed with the demux plugin, then primers were trimmed with the cut adapt tool. Using the DADA2 plugin, the sequence was filtered, combined, and denoised, and the chimera was removed according to [19]. The feature-classifier plugin was used to assign taxonomy to amplicon sequence variants (ASVs) using a classify-sklearn naïve Bayes taxonomic classifier against the SILVA Release 132 Database [20].

The sequence data were arranged using Microsoft Excel 2013^®^ (Microsoft Corporation, Redmond, WA, USA) and analyzed by Origin (v2021). The alpha diversity indices, including the Chao1 and Shannon index, were visualized in the bar graphs. The analysis of beta diversity was carried out to observe the structural variation of the rhizosphere soil microbiome across samples with principal coordinate analysis (PCA) [21]. The difference in the rhizosphere soil microbiota between groups was determined by analysis of the similarities, and the linear discriminant analysis effect size (LEfSe) was carried out using default parameters to observe the differentially abundant taxa between groups [22]. To investigate the impact of environmental factors (such as pH, total N, available P, etc.) on the microbial community structure, redundancy discriminant analysis (RDA) was carried out using Origin (v2021). Furthermore, the SPSS 16.0 software (SPSS Inc., Chicago, IL, USA) was used to calculate the significance test (*p* < 0.05) of the main treatments and their interaction through an analysis of variance (one-way ANOVA) after testing for normality and variance homogeneity.

## 3. Results and Discussion

### 3.1. Effects of Different Microbial Fertilizers on Corn Production

In order to examine the effect of different microbial fertilizers on corn production in newly reclaimed soil, the dry weight of the corn was evaluated 110 days after planting. The result from this study indicated that the weight of air-dried corn ears was affected by three different microbial fertilizers while the effect was dependent on the kind of fertilizer. In detail, compared to the control, there was a significant increase in the weight of air-dried corn ears by TMF (range, 30.19% to 59.33%), CMA (range, 31.39% to 53.43%), SMF (range, 42.84% to 66.19%), SM (range, 42.20% to 72.89%), and CCF (range, 12.00% to 30.94%) from 2019 to 2021, respectively (Table 2).

Consistent with the results of this study, our previous study also showed that plant growth-promoting microorganisms, as inoculants, can enhance plant and abiotic stresses through a wide variety of mechanisms [13,14]. The price of SMF and SM is about 30,000 and 10,000 Chinese yuan/ha, respectively, which is higher than that of chemical fertilizer (about 4500 Chinese yuan/ha); however, this result indicates that microbial fertilizers, particularly SMF and SM, exhibited a greater promotion effect on corn production in newly reclaimed land. On the other hand, the applied amount of microbial fertilizers could be reduced as the soil fertility improves.

### 3.2. Effects of Different Microbial Fertilizers on Soil pH, Organic Matter Content, and Nutrient Elements

Result from this study indicated that the addition of various microbial fertilizers did not significantly affect the pH of corn rhizosphere soil. However, compared with the control, the soil pH was slightly increased by TMF, SMF, SM, and CCF from pH 6.50 to pH 6.60–6.82 in 2019–2021, excluding a slight decrease by CMA and SM in 2019, CMA in 2020, and CCF in 2021, which is in coordination with our previous studies [13,17,23,24]. Furthermore, the soil OMC was significantly increased by all of the test fertilizers (except CMA in 2019) in 2019–2021. Indeed, in 2019, TMF, SMF, and SM caused a 3.22–17.95% increase in the soil OMC compared to the control while the greatest increase was achieved by SM (17.95%, lower than CCF’s 22.81%). TMF, CMA, SMF, and SM caused a 34.92–106.90% and 54.69–116.95% increase in the soil OMC compared to the control in 2020 and 2021, respectively, while SMF achieved the greatest increase, and there was no significant difference in the OMC between CCF and the control in 2020 and 2021 (Table 3). The results from this study revealed that the soil OMC can be improved by biological organic fertilizer. In agreement with the results of this study, a similar observation of an increase in OMC was observed by Ren et al. [17]. Furthermore, other studies have shown that in agroecosystems, soil OMC contributes to vital soil functions such as sustained biological activity, nutrient cycling, and crop productivity and is a key determinant of soil quality [25,26,27]. Therefore, it can be inferred that microbial fertilizers have great potential to improve the soil quality of newly reclaimed soil.

Compared to the control, the total N was slightly reduced by TMF (4.35%) but significantly increased by SMF (28.99%) and SM (33.33%), and slightly increased by CMA (13.04%) and CCF (5.49%), respectively, in 2019. In 2020, TMF, CMA, and SMF caused a significant increase of 30.77%, 18.68%, and 29.67% in the total N, respectively, and there was no significant change in SM and CCF. The total N was significantly increased by TMF (27.62%) and SMF (47.62%), slightly increased by CMA (15.24%) and SM (16.19%), and slightly decreased by CCF (8.57%) in 2021. Compared to the control, the content of available P was significantly increased by all the treatments, with an 8.95–17.02% increase in 2019 (except a 4.34% reduction by SM), a 44.51–64.96% increase in 2020, and a 73.46–154.89% increase in 2021, respectively. In detail, SMF caused the greatest increase in available P in 2019 and 2021, which caused a 2.57% and 27.30% increase compared to CCF. Similarly, compared to the control, the content of available K was significantly increased (3.89–8.46%, 18.81–25.28%, and 22.82–31.57% in 2019, 2020, and 2021, respectively) by all the treatments, and TMF, CMA, and CCF caused a significant increase of 8.46%, 7.045, and 7.09% in 2019, and there was a significant increase by TMF, CMA, SMF, SM, and CCF (25.18%, 18.81%, 21.82%, 22.58%, and 23.57% in 2020, and 27.10%, 22.82%, 25.64, 27.98%, and 31.57% in 2021, respectively) (Table 4).

Compared to the control, the exchangeable Ca was significantly increased by TMF (19.63%), CMA (30.92%), and CCF (37.89%), and slightly decreased by SMF (0.64%) and SM (5.32%) in 2019. There was a significant increase by CMA (23.27%), and there was no significant change by the other treatments in 2020 but there were significant increases by the TMA (19.64%), CMA (23.10%), SMF (317.16%), SM (24.59%), and CCF (22.94%) treatments in 2021. Similarly, compared to the control, the exchangeable K was significantly increased by CCF (15.96%) and slightly increased by TMA (4.26%), SMF (10.64%), and SM (2.13%), but there was a 7.45% decrease by CMA in 2019. There was a significant reduction in TMF (28.57%) and no significant decrease by CMA (12.86%), SMF (14.29%), and CCF (12.86%), and a slight increase in SM (2.86%), respectively (Table 5).

The results indicated that the soil pH, OMC, total N, available P, available K, exchangeable Ca, and exchangeable Mg were differentially affected by fertilizers. The effect was dependent on the soil parameters and the kind of fertilizer. In conclusion, after three years of continuous use of different microbial fertilizers, the application of SMF resulted in the greatest increase in the soil pH, OMC, total N, and exchangeable Ca compared to the other treatments. The available P and K were increased less by SMF than CCF and SM but were increased more by TMF and CMA. Additionally, exchangeable Mg was increased less by SMF than SM but increased by TMF, CMA, and CCF. Furthermore, it is well known that organic matter has been proposed to exhibit a greater effect on microbial communities than other parameters [15,28,29]. Therefore, a greater effect of SMF than TMF and CMA in the improvement of newly reclaimed soil may be inferred in this study.

### 3.3. Effects of Different Microbial Fertilizers on the Microbial Community Diversity

**Figure 2:**
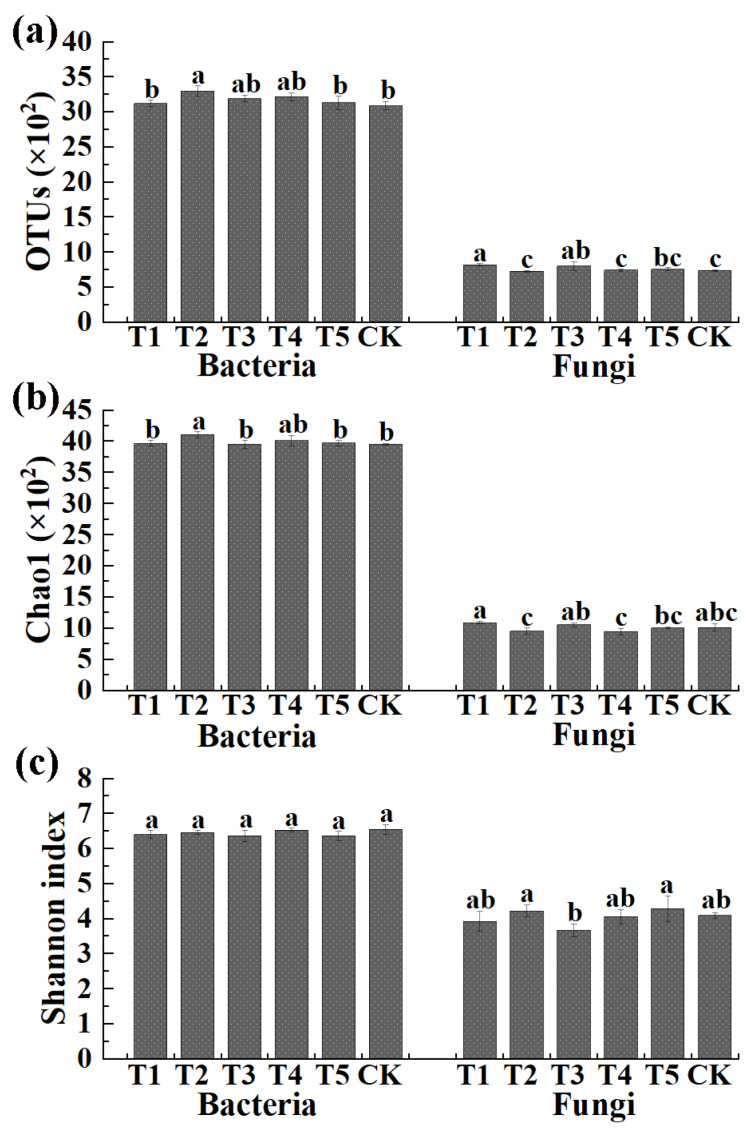
Effects of different microbial fertilizers on the OUT distribution (**a**), the Chao1 index (**b**) and the Shannon’s diversity index (**c**) of bacteria and fungi in corn rhizosphere soil. T1, T2, T3, T4, T5, and CK represents the treatment of Tuzangjin microbial fertilizer (TMF), carbonergic microbial agent (CMA), seaweed microbial fertilizer (SMF), sheep manure (SM), chemical compound fertilizer (CCF), and the control (CK), respectively. Values with different lowercase letters within the same treatments indicate significant differences (*p* < 0.05).

The number of OTUs and the Chao1 and Shannon index in the V3 + V4 region (bacteria) and ITS region (fungi) are shown in Figure 2. Results indicated that the average number of bacterial OTUs was 3122 (3071 to 3162), 3300 (3237 to 3383), 3191 (3154 to 3247), 3219 (3161 to 3274), 3131 (3023 to 3202), and 3088 (3039 to 3155) in TMF, CMA, SMF, SM, CCF, and the control, respectively. Meanwhile, the average Chao1 index and Shannon index of bacteria was 3969 (3940 to 4017), 4109 (4052 to 4141), 3954 (3883 to 4001), 4014 (3932 to 4097), 3971 (3923 to 4008), 3952 (3939 to 3965), 6.40 (6.32 to 6.53), 6.46 (6.40 to 6.52), 6.37 (6.24 to 6.54), 6.53 (6.48 to 6.59), 6.36 (6.21 to 6.44), and 6.55 (6.41 to 6.69) in TMF, CMA, SMF, SM, CCF, and the control, respectively. In general, the bacterial OTUs and Chao1 index were increased (1.10% to 6.87% and 0.05% to 3.97%, respectively) by all five treatments compared to the control, among which CMA caused a significant increase in the OTUs (6.87%) and Chao1 index (3.97%) (Figure 2), but no significant difference was observed in the Shannon index of the bacterial community among all six treatments.

The average number of fungal OTUs was 822 (803 to 838), 728 (713 to 740), 806 (738 to 865), 745 (731 to 761), 759 (735 to 776), and 744 (735 to 757) in TMF, CMA, SMF, SM, CCF, and the control, respectively. Additionally, the average Chao1 index and Shannon index of fungi were 1094 (1066 to 1115), 959 (910 to 1011), 1056 (1025 to 1080), 948 (894 to 990), 1007 (998 to 1020), 1016 (962 to 1079), and 3.92 (3.60 to 4.10), 4.22 (4.10 to 4.41), 3.67 (3.50 to 3.87), 4.06 (3.88 to 4.28), 4.29 (3.95 to 4.67), and 4.09 (4.02 to 4.17) in TMF, CMA, SMF, SM, CCF, and the control, respectively. In general, TMF and SMF caused a significant increase (10.48% and 8.33%, respectively), SM and CCF caused a slight increase (0.13% and 2.02%, respectively), and CMA caused a 2.15% reduction in the fungal OTUs compared to the control, respectively. TMF and SMF caused a slight increase (7.68% and 3.94%, respectively) while CMA, SM, and CCF caused a 5.61%, 6.69%, and 0.89% reduction in the fungal Chao1 index compared to the control, respectively. The Shannon index of fungi was slightly increased by CMA and CCF (3.17% and 4.89%, respectively) and reduced by TMF, SMF, and SM (4.15%, 10.27%, and 0.73%, respectively) compared to the control (Figure 2).

Furthermore, the average bacterial OTUs were 3.80-, 4.53-, 3.96-, 4.32-, 4.13-, and 4.15-fold greater than that of fungi in TMF, CMA, SMF, SM, CCF, and the control, respectively. The bacteria Chao1 indexes were 3.63-, 4.28-, 3.74-, 4.23-, 3.94-, and 3.89-fold greater than those of fungi in TMF, CMA, SMF, SM, CCF, and the control, respectively. Similarly, the bacteria Shannon indexes were 1.63-,1.53-, 1.74-, 1.60-, 1.48-, and 1.60-fold greater than fungi in TMF, CMA, SMF, SM, CCF, and the control, respectively. In addition, CMA caused a greater ratio of bacterial and fungal OUT distribution and diversity indexes compared to CCF. The results indicated that the diversity of bacteria in the rhizosphere soil of corn was significantly increased and CMA significantly reduced the diversity of fungi. Meanwhile, compared to SM, CMA caused a greater ratio of bacterial and fungal OUT distribution but a slightly smaller ratio of diversity indexes, which meant the diversity of fungi in CMA was smaller than that in SM (Figure 2).

In agreement with the results of this study, the role of bio-organic fertilizer in soil microbe has been reported in many previous studies [16,17,30]. For example, the bacterial Chao1 and Shannon indexes of rhizosphere soil were significantly improved by bio-organic fertilizer [30]. The application of bio-organic fertilizer increased the OTUs of bacteria in decline disease bayberry rhizosphere soil [17]. The number of bacteria species in the community increased by 70% after applying bio-organic fertilizer in combination with 30% chemical fertilizer [30].

### 3.4. Effects of Different Microbial Fertilizers on the Soil Microbial Community Structure

**Figure 3:**
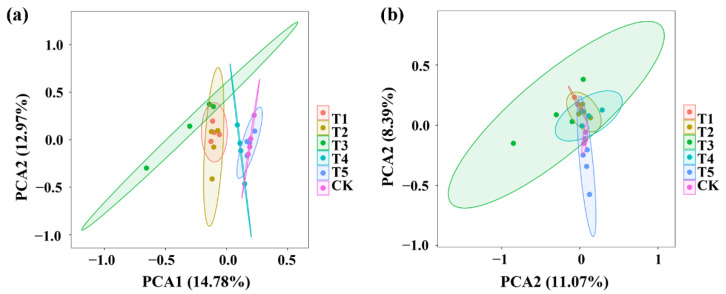
PCA results of soil bacteria (**a**) and fungi (**b**) based on OUT abundance. T1, T2, T3, T4, T5, and CK represent the treatment of Tuzangjin microbial fertilizer (TMF), carbonergic microbial agent (CMA), seaweed microbial fertilizer (SMF), sheep manure (SM), chemical compound fertilizer (CCF), and the control (CK), respectively.

Principal component analysis (PCA) of the bacterial community structure indicated that the four replicates of TMF, CMA, SMF, SM, CCF, and the control were divided into six different groups while TMF, CMA, and SMF were well separated from the treatments of SM, CCF, and the control, indicating that the bacterial community structure of rhizosphere soil was significantly changed by TMF, CMA, and SMF (Figure 3a). Similarly, PCA analysis of the fungal community structure indicated that the four replicates of TMF, CMA, SMF, SM, CCF, and the control were divided into six different groups; however, there was overlap among TMF, CMA, SMF, SM, CCF, and the control. However, TMF and the control were well separated, which indicated that the fungal community structure of rhizosphere soil was significantly changed by TMF (Figure 3b). In general, greater diversity was observed in the four replicates of SMF and CMA in the bacterial community structure, and greater diversity was also observed in the four replicates of SMF in the fungal community structure, respectively (Figure 3). In agreement with the results of this research, continuous application of bio-organic fertilizer caused a significant change in the PCA of the bacterial and fungal communities in barberry and cotton soil [16,30].

**Figure 4:**
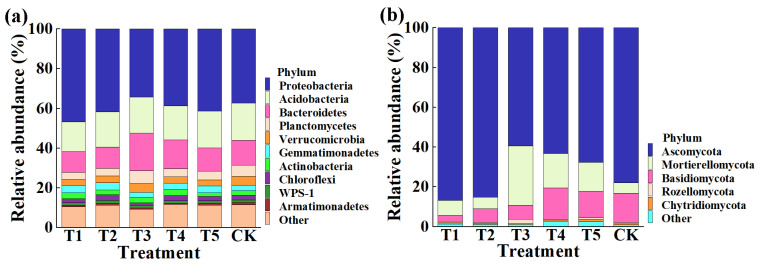
Relative abundance of bacteria (**a**) and fungi (**b**) at the phylum level. T1, T2, T3, T4, T5, and CK represent the treatment of Tuzangjin microbial fertilizer (TMF), carbonergic microbial agent (CMA), seaweed microbial fertilizer (SMF), sheep manure (SM), chemical compound fertilizer (CCF), and the control (CK), respectively.

**Figure 5:**
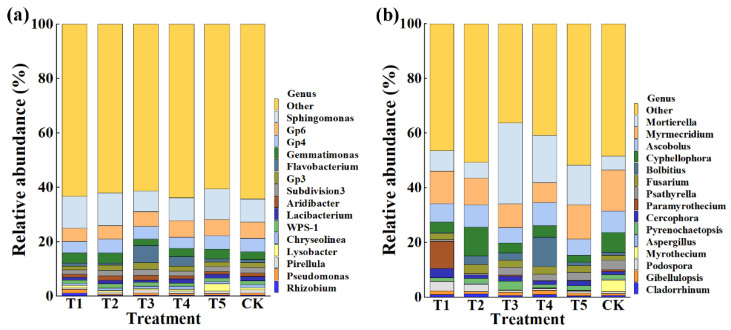
Relative abundance of bacteria (**a**) and fungi (**b**) at the genus level. T1, T2, T3, T4, T5, and CK represent the treatment of Tuzangjin microbial fertilizer (TMF), carbonergic microbial agent (CMA), seaweed microbial fertilizer (SMF), sheep manure (SM), chemical compound fertilizer (CCF), and the control (CK), respectively.

This result indicated that the application of TMF, CMA, SMF, SM, and CCF resulted in a significant change in the composition of the bacterial and fungal community at the phylum (Figure 4) and genus levels compared to the control (Figure 5). Indeed, the top 10 phylum in the rhizosphere soil of corn were selected to generate a relative abundance histogram, in which Proteobacteria, Acidobacteria, Bacteroidetes, Planctomycetes, and Verrucomicrobia were the main bacterial phylum, with a relative abundance of 34.23–46.75%, 15.00–18.74%, 10.47–18.84%, 3.61–6.51%, and 3.08–4.47%, respectively (Figure 4a). Furthermore, the relative abundance histogram based on the top 15 species showed that *Sphingomonas*, *Gp6*, *Gp4*, *Gemmatimonas*, *Gp3*, *Subdivision3*, *Aridibacter*, and *WPS-1* were the main bacterial genes (average relative abundance > 1%). Compared with that of the control, the relative abundance of *Sphingomonas* was increased by 37.11%, 40.06%, and 31.81% in TMF, CMA, and CCF but reduced by 11.76% and 1.50% in SMF and SM, respectively. The relative abundance of *Gp6* was reduced by 17.33%, 13.65%, and 6.36% in TMF, CMA, and SMF but increased by 4.57% and 1.09% in SM and CCF, respectively. The relative abundance of *GP4* was reduced by 16.93%, 7.92%, 19.60%, and 0.13% in TMF, SMF, SM, and CCF, respectively, but increased by 1.41% in CMA. The relative abundance of *Gemmatimonas* was increased by 28.66%, 29.35%, 6.31%, and 19.26% in TMF, CMA, SM, and CCF, respectively, but reduced by 16.40% in SMF. The relative abundance of *GP3* was reduced by 20.95% and 2.79% in TMF and CCF but increased by 7.36%, 33.03%, and 5.27% in CMA, SMF, and SM, respectively. The relative abundance of *Subdivision3* was reduced by 23.26%, 3.09%, 23.26%, and 17.46% in TMF, CMA, SM, and CCF, respectively, but increased by 7.14% in SMF. The relative abundance of *Aridibacter* and *WPS-1* was reduced by 8.78%, 6.30%, 12.03%, 5.95%, 11.24%, and 10.13% in TMF, SM, and CCF, respectively, but increased by 32.30%, 35.08%, 12.26%, and 4.22% in CMA and SMF, respectively (Figure 5a). In particular, more attention should be paid to *Sphingomonas* and *Gemmatimonas*. *Sphingomonas* has been reported to play a role in plant growth by producing plant growth hormones, e.g., gibberellins and indole acetic acid, during stress conditions such as drought, salinity, and heavy metals in agricultural soil [31]. Studies have revealed that *Gemmatimonas* can reduce the potent greenhouse gas N_2_O under both anaerobic and aerobic conditions [32,33].

According to the distribution and relative abundances of fungi in corn rhizosphere soil at the phylum level, Ascomycota, Mortierellomycota, and Basidiomycota were the main fungal phyla, with a relative abundance of 59.48–86.85%, 5.11–29.71%, and 2.94–15.79% in TMF, CMA, SMF, SM, CCF, and the control, respectively (Figure 4b). Furthermore, the relative abundance histogram based on the top 15 species showed that *Mortierella*, *Myrmecridium*, *Ascobolus*, *Cyphellophora*, *Fusarium*, *Cercophora*, and *Pyrenochaetopsis* were the main bacterial genes (average relative abundance > 1%). Compared with that of the control, TMF, CMA, SMF, SM, and CCF caused a 47.82%, 12.50%, 482.47%, 239.43%, and 183.18% increase in the relative abundance of *Mortierella*; a 33.12%, 97.65%, 50.87%, 66.75%, and 41.44% increase in the relative abundance of *Fusarium*; a 237.65%, 18.85%, 66.05%, 40.15%, and 78.30% increase in the relative abundance of *Cercophora*; and a 20.64%, 34.64%, 42.19%, 51.53%, and 15.24% reduction in the relative abundance of *Myrmecridium*, respectively. Interestingly, the relative abundance of *Ascobolus* was reduced by 14.73%, 30.35%, and 25.37% in TMF, SMF, and CCF but increased by 1.89% and 7.13% in CMA and SM, respectively. The relative abundance of *Cyphellophora* was reduced by 46.10%, 48.53%, 41.17%, and 65.74% in TMF, SMF, SM, and CCF, respectively, but increased by 47.87% in CMA. The relative abundance of *Pyrenochaetopsis* was reduced by 31.16% and 31.55% in TMF and SM but increased by 11.66%, 99.72%, and 3.07% in CMA, SMF, and CCF, respectively (Figure 5b). Studies have revealed that *Mortierella* in the rhizophore of wheat is significantly associated with an increase in crop growth and yield [34]. The increase in the relative abundance of *Mortierella* may promote plant growth.

### 3.5. Effects of Different Microbial Fertilizers on the Rhizosphere Microbiome and Biomarkers

**Figure 6:**
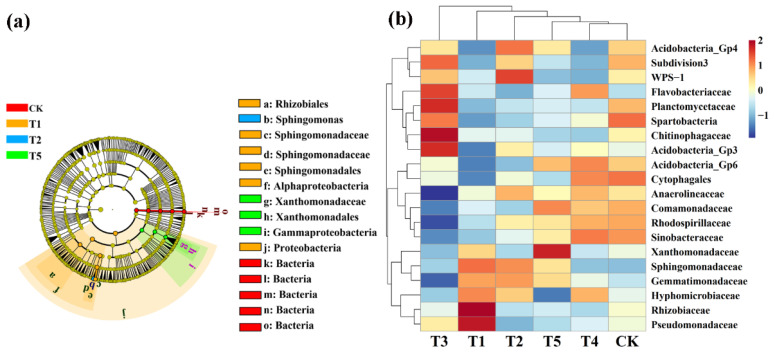
Linear discriminant analysis (LDA) effect size (LeFse) of the bacterial taxa (**a**), which identifies the most differentially abundant taxa among the different microbial fertilizer treatments. Only taxa with LDA values greater than 4 (*p* < 0.05) are shown. Hierarchical clustering analysis and heat mat at the family level (**b**). The tree plot represents a clustering analysis of the top 20 bacteria at the family level according to their Person correlation coefficient matrix and relative abundance; the upper tree plot represents a clustering analysis of soil samples according to the Euclidean distance of data. T1, T2, T3, T4, T5, and CK represent the treatment of Tuzangjin microbial fertilizer (TMF), carbonergic microbial agent (CMA), seaweed microbial fertilizer (SMF), sheep manure (SM), chemical compound fertilizer (CCF), and the control (CK), respectively.

LeFse (LDA > 4, *p* < 0.05) was used to reveal the biomarkers with the largest difference between the rhizosphere soil microbial communities of corn under different microbial fertilizers treatments. A total of 15 bacterial biomarkers were found in all six treatments (Figure 6a), and the control mainly identified six types of *bacteria*. TMF is enriched with *Rhizobiales*, *Sphingomonadaceae*, *Sphingomonadales*, *Alphaproteobacteria*, and *Proteobacteria*. CMA is enriched with *Sphingomonas* while CCF is rich in *Xanthomonadaceae*, *Xanthomonadales*, and *Gammaproteobacteria*. We further visually explained the difference in the rhizosphere bacterial community’s relative abundance composition (family level) under six fertilizer conditions through heat maps (Figure 6b). The microbial fertilizer treatment enriched *Pseudomonadaceae*, *Rhizobiaceae*, *Acidobacteria_Gp3*, *Chitinophagaceae*, *Planctomycetaceae*, *WPS-1*, and *Flavobacteriaceae* (*p* < 0.05). *Acidobacteria_Gp6*, *Cytophagales*, *Comamonadaceae*, *Rhodospirillaceae*, and *Sinobacteaceae* were significantly reduced (*p* < 0.05).

**Figure 7:**
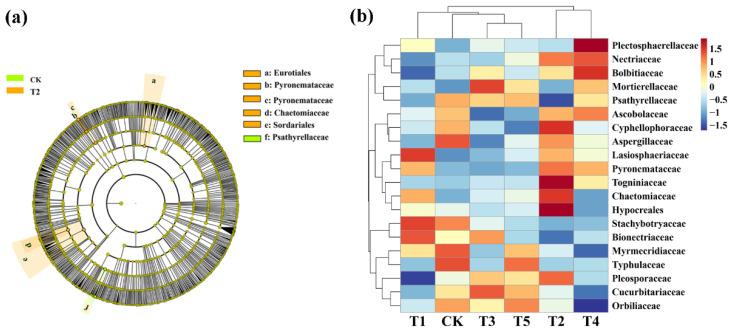
Linear discriminant analysis (LDA) effect size (LeFse) of the fungal taxa (**a**), which identified the most differentially abundant taxa among the different microbial fertilizer treatments. Only taxa with LDA values greater than 4 (*p* < 0.05) are shown. Hierarchical clustering analysis and heat mat at the family level (**b**). The tree plot represents a clustering analysis of the top 20 fungi at the family level according to their Person correlation coefficient matrix and relative abundance; the upper tree plot represents a clustering analysis of soil samples according to the Euclidean distance of data. T1, T2, T3, T4, T5, and CK represent the treatment of Tuzangjin microbial fertilizer (TMF), carbonergic microbial agent (CMA), seaweed microbial fertilizer (SMF), sheep manure (SM), chemical compound fertilizer (CCF), and the control (CK), respectively.

Meanwhile, LeFse was also executed at LDA > 4 (*p* < 0.05) in rhizosphere soil fungal communities under different treatment conditions. CMA identified five types: *Eurotiales*, *Pyronemataceae*, *Pyronemataceae*, *Chaetomiaceae*, and *Sordariales* while the control is rich in *Psathyrellaceae* (Figure 7a). We also further visually explained the difference in the relative abundance composition (family level) of the rhizosphere fungal community under the six fertilizer conditions through heat maps (Figure 7b). The microbial fertilizer treatment enriched *Nectriaceae*, *Mortierellaceae*, *Cyphellophoraceae*, *Lasioshaeriaceae*, *Pyronemataceae*, *Togniniacece*, *Chaetomiaceae*, and *Hypocreales* (*p* < 0.05). *Myrmecridiaceae*, *Typhulaceae*, and *Orbilibceae* were significantly reduced (*p* < 0.05). The results demonstrate that microbial fertilizers can increase or reduce the existence of certain specific species that change the community structure of bacteria in rhizosphere soil of corn.

### 3.6. Effects of Different Microbial Fertilizers on RDA of Soil Properties and Microbial Communities

**Figure 8:**
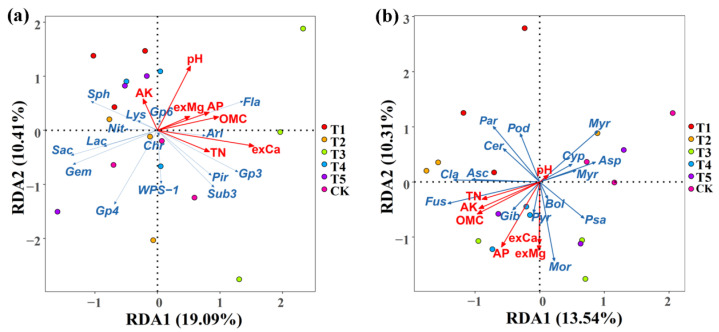
Redundancy discriminant analysis (RDA) of the rhizosphere bacterial (**a**) and fungal (**b**) community compositions at the genus level with the soil physicochemical properties. Sph: Sphingomonas; Gem: Gemmatimonas; Fla: Flavobacterium; Sub: Subdivision3; Ari: Aridibacter; Lac: Lacibacterium; Chr: Chryseolinea; Lys: Lysobacter; Pir: Pirellula; Pse: Pseudomonas; Rhi: Rhizobium. Mor: Mortierella; Myrm: Myrmecridium; Asc: Ascobolus; Cyp: Cyphellophora; Bol: Bolbitius; Fus: Fusarium; Psa: Psathyrella; Par: Paramyrothecium; Cer: Cercophora; Asp: Aspergillus; Pye: Pyrenochaetopsis; Pod: Podospora; Myr: Myrothecium; Cla: Cladorrhinum; Gib: Cladorrhinum. OMC: organic matter contain; TN: total N; AP: available P; AK: available K; exCa: exchangeable Ca; exMg: exchangeable Mg. T1, T2, T3, T4, T5, and CK represent the treatment of Tuzangjin microbial fertilizer (TMF), carbonergic microbial agent (CMA), seaweed microbial fertilizer (SMF), sheep manure (SM), chemical compound fertilizer (CCF), and the control (CK), respectively.

The association of the soil properties with the rhizosphere microbial community composition was examined using a redundancy discriminant analysis (RDA), which has been used previously to explore the relationship between environmental factors and microbial communities. Results from this study showed a total of 29.50% and 23.84% of the cumulative variance of the rhizosphere microbial community–factor correction at the bacterial (Figure 8a) and fungal (Figure 8b) genus level, respectively. The contributions of the five main variables: exchangeable Ca, pH, organic matter content, total N, and available P, explained 69.89%, 33.17%, 28.82%, 22.57%, and 21.82% of the bacterial community at the genus level, respectively (Figure 8a, Table 6), while the contributions of the six main variables: available P, exchangeable Mg, organic matter content, exchangeable Ca, available K, and total N, explained 39.44%, 35.62%, 30.38%, 29.30%, 26.95%, and 22.00% of the fungal community at the genus level, respectively (Figure 8b, Table 6).

Furthermore, this study showed a complex relationship between soil nutrient elements and microbial growth because the compositions of the bacterial and fungal communities in corn rhizosphere soil were significantly affected by the different soil properties. In agreement with the result of this study, previous studies also found that the growth of soil microbes was affected by many environmental factors, such as pH, organic matter content, nitrogen, and available P [3,17].

## 4. Conclusions

This study showed that microbial fertilizer, especially SMF, had a better effect on improving the soil organic matter content and some soil nutrient elements (such as total N, exchangeable Ca) than CCF. Furthermore, the composition of the microbial communities in corn rhizosphere soil was significantly affected by exchangeable Ca, organic matter content, total N, and available P at the genus level. Compared with the control, SMF caused a significant increase in the relative abundance of *Flavobacteriaceae*, *Planctomycetaceae*, *Chitinophagaceae*, *Acidobacteria_Gp3*, and *Mortierellaceae*, and a reduction in the relative abundance of *Cytophagales*, *Comamonadaceae*, *Rhodospirillaceae*, *Sinobacteaceae*, *Aspergillaceae*, *Myrmecridiaceae*, and *Typhulaceae* while the relative abundance of *Xanthomonadaceae* and *Mortierellaceae* increased but *Cytophagales*, *Spartobacteria*, and *Cyphellophoraceae* were reduced by CCF. Overall, the results of this study revealed significant changes under different fertilizer conditions in the microbiota and chemical properties of corn soil. Our results suggest that microbial fertilizers, particularly SMF and SM, can be used as good amendments for newly reclaimed land.

## Figures and Tables

**Table 1:** The composition of each fertilizer used in this study.

Fertilizer	OMC (%)	NPK Content (%)	MS Content (million/g)	Others
TMF	80	5.0	2.0	humic acid, medium, and trace elements
CMA	45	-	5.0	biocarbon, fulvic acid, humic acid
SMF	50	5.0	4.0	seaweed protein, and polysaccharide, organic Ca (1.0%)
SM	30	4.0	-	-
CCF	-	48.0	-	NO_3_ and K_2_SO_4_

TMF: Tuzangjin microbial fertilizer; CMA: carbonergic microbial agent; SMF: seaweed microbial fertilizer; SM: sheep manure; CCF: chemical compound fertilizer; OMC: organic matter content; MS: microbe content.

**Table 2:** Effects of different microbial fertilizers on corn production.

Treatments	Weight of Air-Dried Corn Ears (kg)					
	2019	GPE%	2020	GPE%	2021	GPE%
TMF	14.10 ± 1.23	30.19 ab	15.47 ± 1.57	52.63 ab	16.65 ± 2.09	59.33 ab
CMA	14.23 ± 1.01	31.39 ab	15.30 ± 1.83	50.99 bc	16.03 ± 0.32	53.43 ab
SMF	15.47 ± 2.67	42.84 a	18.63 ± 2.05	71.05 ab	17.37 ± 2.15	66.19 a
SM	15.40 ± 2.70	42.20 a	17.33 ± 0.86	83.88 a	18.07 ± 2.28	72.89 a
CCF	12.13 ± 0.40	12.00 b	12.23 ± 0.75	20.72 c	13.68 ± 2.18	30.94 b
Control	10.83 ± 0.45	-	10.13 ± 0.72	-	10.45 ± 1.67	-

TMF: Tuzangjin microbial fertilizer; CMA: carbonergic microbial agent; SMF: seaweed microbial fertilizer; SM: sheep manure; CCF: chemical compound fertilizer. The growth promotion efficacy (GPE%) = (treatment − control)/control × 100%. Data were presented as mean ± standard deviation. Values with different lowercase letters within the same columns indicate significant differences (*p* < 0.05).

**Table 3:** Effects of different microbial fertilizers on the pH and organic matter content in newly reclaimed soil.

Treatments (kg/m^2^)	pH			OMC (g/kg)		
	2019	2020	2021	2019	2020	2021
TMF	6.63 ± 0.05 a	6.61 ± 0.10 ab	6.74 ± 0.15 a	14.07 ± 1.42 bc	18.07 ± 0.95 c	22.87 ± 1.18 b
CMA	6.40 ± 0.54 a	6.47 ± 0.13 b	6.60 ± 0.30 a	12.37 ± 0.46 c	17.00 ± 1.80 c	19.80 ± 1.61 c
SMF	6.66 ± 0.07 a	6.82 ± 0.13 a	6.73 ± 0.07 a	13.80 ± 1.31 bc	26.07 ± 1.12 a	27.77 ± 1.75 a
SM	6.47 ± 0.51 a	6.61 ± 0.2 ab	6.81 ± 0.05 a	15.77 ± 1.27 ab	22.23 ± 0.40 b	24.97 ± 1.16 b
CCF	6.65 ± 0.18 a	6.60 ± 0.10 ab	6.50 ± 0.17 a	16.42 ± 1.32 a	13.33 ± 1.19 d	13.17 ± 0.68 d
Control	6.50 ± 0.09 a	6.53 ± 0.21 ab	6.55 ± 0.16 a	13.37 ± 0.97 c	12.60 ± 1.00 d	12.80 ± 0.53 d

TMF: Tuzangjin microbial fertilizer; CMA: carbonergic microbial agent; SMF: seaweed microbial fertilizer; SM: sheep manure; CCF: chemical compound fertilizer; OMC: organic matter content. Data were presented as mean ± standard deviation. Values with different lowercase letters within the same columns indicate significant differences (*p* < 0.05).

**Table 4:** Effects of different microbial fertilizers on the total N, available P, and available K in newly reclaimed soil.

Treatments (kg/m^2^)	Total N (g/kg)			Available P (mg/kg)			Available K (mg/kg)		
	2019	2020	2021	2019	2020	2021	2019	2020	2021
TMF	0.66 ± 0.04 c	1.19 ± 0.07 a	1.34 ± 0.10 ab	17.13 ± 0.84 ab	18.40 ± 0.94 b	23.07 ± 1.19 d	396.10 ± 12.36 a	422.64 ± 15.10 a	424.65 ± 13.97 ab
CMA	0.78 ± 0.04 bc	1.08 ± 0.05 ab	1.21 ± 0.13 bc	16.32 ± 1.56 abc	18.45 ± 0.10 b	23.90 ± 0.35 d	390.93 ± 13.61 a	401.15 ± 14.53 b	410.33 ± 11.40 b
SMF	0.89 ± 0.07 ab	1.18 ± 0.07 a	1.55 ± 0.26 a	17.53 ± 1.41 a	19.53 ± 0.76 ab	33.90 ± 1.06 a	383.58 ± 17.44 ab	411.31 ± 3.58 ab	419.77 ± 5.21 b
SM	0.92 ± 0.13 a	0.97 ± 0.12 bc	1.22 ± 0.07 bc	14.33 ± 0.90 c	18.02 ± 1.09 b	29.67 ± 0.97 b	379.41 ± 3.26 ab	413.85 ± 11.09 ab	427.57 ± 6.08 ab
CCF	0.73 ± 0.02 c	0.96 ± 0.09 bc	0.96 ± 0.13 d	17.09 ± 1.13 ab	20.57 ± 1.25 a	26.63 ± 0.68 c	391.10 ±15.61 a	417.22 ± 3.66 a	439.59 ± 11.71 a
Control	0.69 ± 0.10 c	0.91 ± 0.04 c	1.05 ± 0.04 cd	14.98 ± 0.47 bc	12.47 ± 0.67 c	13.3 ± 0.61 e	365.21 ± 10.06 b	337.63 ± 3.19 c	334.10 ± 3.25 c

TMF: Tuzangjin microbial fertilizer; CMA: carbonergic microbial agent; SMF: seaweed microbial fertilizer; SM: sheep manure; CCF: chemical compound fertilizer. Data were presented as mean ± standard deviation. Values with different lowercase letters within the same columns indicate significant differences (*p* < 0.05).

**Table 5:** Effects of different microbial fertilizers on the exchangeable Ca and exchangeable Mg in newly reclaimed soil.

Treatments (kg/m^2^)	Exchangeable Ca (cmol (1/2 Ca^2+^)/kg)			Exchangeable Mg (cmol (1/2 Mg^2+^)/kg)		
	2019	2020	2021	2019	2020	2021
TMF	13.04 ± 1.01 b	12.64 ± 0.20 ab	7.25 ± 0.50 b	0.98 ± 0.04 abc	0.50 ± 0.02 c	0.97 ± 0.08 b
CMA	14.27 ± 0.91 ab	13.93 ± 0.96 a	7.46 ± 0.70 b	0.87 ± 0.08 c	0.61 ± 0.05 bc	1.02 ± 0.04 ab
SMF	10.83 ± 0.65 c	11.23 ± 0.59 b	25.28 ± 0.68 a	1.04 ± 0.06 ab	0.60 ± 0.02 bc	1.11 ± 0.07 ab
SM	10.32 ± 0.17 c	11.57 ± 0.14 b	7.55 ± 0.56 b	0.96 ± 0.12 abc	0.72 ± 0.03 a	1.17 ± 0.11 a
CCF	15.03 ± 1.47 a	11.76 ± 0.98 b	7.45 ± 0.65 b	1.09 ± 0.05 a	0.61 ± 0.03 b	0.95 ± 0.11 b
Control	10.90 ± 1.17 c	11.30 ± 1.31 b	6.06 ± 0.26 c	0.94 ± 0.03 bc	0.70 ± 0.13 ab	1.05 ± 0.06 ab

TMF: Tuzangjin microbial fertilizer; CMA: carbonergic microbial agent; SMF: seaweed microbial fertilizer; SM: sheep manure; CCF: chemical compound fertilizer. Data were presented as mean ± standard deviation. Values with different lowercase letters within the same columns indicate significant differences (*p* < 0.05).

**Table 6:** Contribution of the soil environment to bacteria and fungi taxa at the genus level.

Soil Environment	Contribution at the Bacterial Genus Level (%)	Contribution at the Fungal Genus Level (%)
pH	33.17	0.80
Organic matter content	28.82	30.38
Total N	22.57	22.00
Available P	21.82	39.44
Available K	7.37	26.95
Exchangeable Ca	69.89	29.30
Exchangeable Mg	8.96	35.62

## Data Availability

All data supporting the conclusions of this article are included in this article.
